# Guidance on the severity classification of scientific procedures involving fish: report of a Working Group appointed by the Norwegian Consensus-Platform for the Replacement, Reduction and Refinement of animal experiments (Norecopa)

**DOI:** 10.1258/la.2011.010181

**Published:** 2011-10

**Authors:** P Hawkins, N Dennison, G Goodman, S Hetherington, S Llywelyn-Jones, K Ryder, A J Smith

**Affiliations:** 1 Research Animals Department, RSPCA, Wilberforce Way, Southwater, West Sussex RH13 9RS, UK; 2 Animals (Scientific Procedures) Inspectorate, Home Office, PO Box 6779, Dundee DD1 9WW, UK; 3 Biological Services, The University of Edinburgh, Chancellor Building, 49, Little France Crescent, Edinburgh EH16 4SB, UK; 4 CEFAS, Pakefield Road, Lowestoft, NR33 0HT, UK; 5 King's College London, Biological Services Unit, 4th floor, Hodgkin Building, Guy's Campus, London SE1 1UL, UK; 6 Norecopa, c/o Norwegian Veterinary Institute, PO Box 750 Sentrum, N-0106 Oslo, Norway

**Keywords:** Fish, harm–benefit assessment, humane endpoints, refinement, severity

## Abstract

The severity classification of procedures using animals is an important tool to help focus the implementation of refinement and to assist in reporting the application of the 3Rs (replacement, reduction and refinement). The recently revised Directive that regulates animal research and testing within the European Union requires Member States to ensure that all procedures are classified as ‘non-recovery’, ‘mild’, ‘moderate’ or ‘severe’, using assignment criteria set out by the European Commission (EC). However, these are focused upon terrestrial species, so are of limited relevance to fish users. A Working Group set up by the Norwegian Consensus-Platform for the 3Rs (Norecopa) has produced guidance on the classification of severity in scientific procedures involving fish, including examples of ‘subthreshold’, ‘mild’, ‘moderate’, ‘severe’ and ‘upper threshold’ procedures. The aims are to complement the EC guidelines and help to ensure that suffering in fish is effectively predicted and minimized. Norecopa has established a website (www.norecopa.no/categories) where more information on severity classification for procedures using fish, including field research, will be made available.

## Background

An effective prediction of the effects of a research protocol on the animals concerned helps to ensure that any pain, suffering or distress they may experience will be effectively anticipated, recognized and alleviated. This is essential not only for animal welfare but also for scientific validity, because physiological and behavioural responses to suffering can significantly affect data quality. Severity classification is thus an important tool to help focus the implementation of refinement, including monitoring its progress, and to assist in reporting the application of the 3Rs (replacement, reduction and refinement) of Russell and Burch,^1^ which is now an integral part of the legislation on animal research and testing in many countries. Predictions of severity are also fundamental to the harm–benefit assessments undertaken by bodies such as regulatory authorities and ethical committees when deciding whether or not a project should be licensed or funded.

There may also be a legal requirement to predict and classify severity. For example, the new Directive regulating animal use within the European Union, which must be implemented within all Member States by January 2013, requires the severity of each procedure to be classified on the basis of the ‘degree of pain, suffering, distress or lasting harm expected to be experienced by an individual animal during the course of the procedure’, with the aim of enhancing transparency, facilitating the project authorization process and providing tools for monitoring compliance.^2^ Member States will have to ensure that all procedures are classified as ‘non-recovery’, ‘mild’, ‘moderate’ or ‘severe’ on a case-by-case basis, using the assignment criteria set out in a European Commission (EC) Working Group report on severity classification.^3^ This focuses heavily on procedures that are relevant to terrestrial laboratory animal species.

One of the activities of Norecopa, Norway's Consensus-Platform for the Replacement, Reduction and Refinement of animal experiments, is to arrange international consensus meetings on harmonization of the care and use of animals in research. At a Norecopa meeting in September 2009, the participants produced a consensus statement describing actions that should be taken to advance the welfare of fish in research.^4^ Norecopa subsequently produced a list of tasks needed to increase the implementation of the 3Rs in fish research.^5^ One of these tasks was to produce the present document, which aims to complement the EC report on severity classification by providing examples of scientific procedures using fish. These are listed in Tables 1–5. Some relevant examples from the EC report, with comments where appropriate, have been included in the tables, to aid comparison. Norecopa has established a website (www.norecopa.no/categories) where more information on severity classification for procedures using fish, including field research, will be made available.

**Table 1:** Subthreshold procedures using fish

Behavioural studies that do not involve any other regulated procedures, for example observation of choice of shelter.
Exposure to an artificial predator where escape into a refuge is immediately possible.
Feeding studies where food restriction does not cause any harm. It should be noted that this is a more complex issue for fish than it is for many mammals, where a differential of no more than 15% has been accepted as below threshold by some authorities previously.^17^ In fish, significant levels of weight loss are highly species- and life stage-specific, as some species will naturally stop feeding at some stages of their life cycle (such as spawning) and may experience extreme weight loss and poor body condition. Likewise, fish may naturally exhibit periods of extreme food intake and subsequent weight gain. Food restriction is likely to be more stressful to farmed fish that have been selected for rapid growth rates. With these provisos, a subthreshold food restriction study would be where the weight loss (adult), or reduction in weight gain (larval and juvenile forms), is not sufficiently different to cause consequences for health or welfare to that of non-deprived fish matched for age, sex and physiological state.
Withdrawal of food for a short interval relative to normal food intake at that stage of the life cycle, for example food withdrawal in adult salmonids for up to 48 h.
Marking using non-toxic and non-aversive dyes in the water (e.g. tetracycline-based dyes for otolith tagging).
Manipulations of photoperiod, for example to delay or accelerate maturation where similar protocols have been shown previously not to cause significant harm.
Manipulations of temperature within temperature ranges experienced by the species in its natural habitat where the speed of change is such that the animals can adapt without significant physiological stress.
Manipulations of water gases and ion levels that are within ranges experienced and tolerated by the species in its natural habitat where such changes occur gradually.
**Examples from the EC Working Group, with comments on their applicability to fish**
*Non-invasive observation of normal behaviour without disturbing the animal.*
*Open field testing.*
*Adding inert markers in the diet to follow passage of digesta.*
*Feeding a diet that meets the full nutritional needs of the animals.*
Comment: such studies should be classified as mild rather than subthreshold if they include weighing and measuring under anaesthesia, and the animals would not undergo this procedure at a similar frequency as part of routine husbandry (such as grading of fish by weight to separate them into appropriate weight classes, to optimize feeding regimes or growth).
*Breeding genetically altered animals that are expected to have no clinically detectable adverse phenotype.*

## Special considerations for fish species

Classification of the severity of procedures when using fish can be problematic for a number of reasons.

### Removal of fish from water can increase severity

Many protocols involve catching and handling fish, which in itself can be difficult, and some protocols involve exposing fish to air. Although fish are routinely removed from water for many procedures (such as vaccination) without complications, the possibility of injury should always be considered. Removal from water can cause complications such as scale loss or gill collapse and can cause stress even in the absence of any physical injury. This can add to the overall severity of the procedure, with both scientific and animal welfare implications.^6–9^ For example, the results of toxicological trials have been affected by the degree of handling stress and disturbance experienced by fish.^10^

It is good practice to avoid handling fish, or to handle them in containers to avoid exposure to air, where possible.^11^ Handling could be refined by using sedation or anaesthesia, noting that some anaesthetic and sedative agents may be aversive, so that any potential benefits of using these would need to be considered against the stress caused to the fish. Ideally, the anaesthetic or sedative would be added to the home tank to avoid handling, although this may not always be practicable.

The Working Group suggests that it may be possible to reduce the negative impact of stressors, such as netting, by training the fish to associate them with positive events. For example, one approach could be to offer a food reward as the fish enters the net, delaying routine feeding until after this procedure if necessary.

### The severity of many procedures is species-specific within fish

There are over 25,000 species of fish, living in a wide range of habitats. To treat ‘fish’ as one group is probably even less meaningful than to attempt to produce guidelines for ‘mammals’. As fish are the most diverse of all vertebrate classes, it is highly likely that the impact of a given procedure will vary between species. Criteria for the categorization of severity in fish within individual projects should thus address species-specific and individual variations in the suffering associated with procedures and other stressful events such as capture, handling and immobilization, particularly when procedures are performed out of water.

In addition to the high degree of interspecific variation, many species of fish undergo large physiological changes as a natural part of their life cycle. This may well mean that the same procedure will affect different age groups in different ways. For all of these reasons, this report can only provide general guidance and should be interpreted in conjunction with specialist advice on each species. There is unfortunately a lack of species-specific guidelines on refining fish care and use at present; however, this should not be used as an excuse to accept lower standards than those that would be applied to terrestrial vertebrates.

### The detection and alleviation of pain, suffering and distress in fish can be problematic

A recent report identifying knowledge gaps relating to the welfare of fish used in research highlighted the need for better indicators of pain, suffering and distress.^12^ Currently available indicators include clinical signs in individual fish (for example, respiratory rate, food consumption and health status) and signs of stress in groups of fish (such as social behaviour and activity level). These indicators should be observed and recorded daily.^11^

Techniques to manage pain in fish are in their infancy and are largely based on extrapolations from protocols developed for use in mammals. There is an urgent need to develop effective methods to detect and alleviate suffering for each of the commonly used species. Better indicators would not only facilitate the correct prediction and classification of severity, but would also help to ensure adequate pain alleviation, which could allow the transfer of a procedure to a milder severity class.

### Societal perceptions of fish can vary

There has traditionally been more tolerance of stress, disease and mortality as an endpoint in fish research, compared with research using mammals, which probably reflects general attitudes to fish in society.^13^ Any such attitudes should be challenged within a research setting.

### Mortality rates in fish can be naturally high

Assessment of the severity of a procedure in fish is complicated by the fact that high mortality rates are often observed in natural populations, particularly during the developmental stages. This may sometimes make it difficult to distinguish deaths caused by the experiment from natural mortality, although mortality of adult fish is more likely to be a result of poor techniques or husbandry standards than is larval mortality.

## Severity classification for procedures using fish

The examples in Tables 1–5 follow the classifications used in the EC report. Those that are identical to (or closely resemble) examples given in the EC report are grouped together in the bottom half of the tables.

When considering these examples, it is important to note that it is assumed that all procedures, including capture and handling, are fully refined and performed optimally by competent persons. Also, assessment of severity should include an overall assessment of the *total* harm or distress produced by a procedure or study, that is, the cumulative suffering caused by potentially distressing elements. For example, factors such as water quality parameters, handling techniques and husbandry can all affect cumulative severity. Repeated procedures that are not particularly invasive or even below threshold may, over a period of time, result in significant suffering for the animal concerned; particularly if they are not given sufficient time to recover between them. This is especially relevant for fish where procedures involve removal from water, for example repeated blood sampling may result in greater cumulative suffering for fish than mammals. All of this should be borne in mind, especially when considering the procedures classified in this document as ‘mild’ (see Table 2). Other points to note are that the duration of a procedure is relevant to its severity classification, and changes in the definition and application of an endpoint may move a procedure from one category to another.

**Table 2:** Mild procedures using fish

Behavioural studies involving short-term exposure to an artificial predator and where escape is not possible.
Research into some diseases, where humane endpoints are applied at the first clinical sign of disease or earlier. Note that the severity of the clinical signs will vary with the severity of the disease.
Feeding studies where there is no reduction in quantity or quality of the diet compared with normal feed, but which involve weighing and measuring under anaesthesia, in excess of that required for husbandry purposes.
Gentle handling of fish out of water, including the administration of an injection or the use of a minimally invasive identification method, where removal from water is brief.
Induction and maintenance of anaesthesia using a route and agent appropriate to the species and life stage, for example to weigh and measure fish for a scientific purpose.
Blood sampling under anaesthesia where volumes and techniques are limited to those recommended by published guidelines and/or national legislation.
Removal of a small part of one fin of fish where rapid healing is expected. The effect of fin clipping will, however, depend upon several factors including the functional importance of the fin and/or its innervation.
Gastric lavage under anaesthesia.
Forcible removal of a small number of scales for genotyping or age determination.
Maintenance of external parasites on host fish where clinical or behavioural signs are minor or transitory.
Insertion of a telemetry device into the stomach by oral gavage under general anaesthesia, where the weight, shape or volume of the device is not expected to have a significant effect on physiological function.
Toxicological studies where animals are humanely killed at or before onset of clinical signs. As with applying humane endpoints in disease studies, some agents may cause severe adverse effects without clinical signs being identified, so this may not apply in the case of highly toxic agents.
**Examples from the EC Working Group, with comments on their applicability to fish**
*Short-term (<24 h) restraint in metabolic cages.*
Comment: This translates as short-term restriction of movement for fish.
*Studies involving short-term deprivation of social partners, short-term solitary housing of sociable species.*
*Models which expose animals to noxious stimuli which are briefly associated with mild pain, suffering or distress, and which the animals can successfully avoid.*
*Feeding of modified diets that do not meet all of the animal's nutritional needs and are expected to cause mild clinical abnormality within the time-scale of the study.*
*Breeding of genetically altered animals which is expected to result in a phenotype with mild effects.*
*Pharmacokinetic studies where a single dose is administered and a limited number of blood samples taken (totalling <10% of circulating volume) and the substance is not expected to cause any detectable adverse effect.*
Comment: Little is known about total blood volumes in fish, so the Working Group suggests a general limit of 1 mL/kg as recommended by the Canadian Council on Animal Care (CCAC).^18^
*Non-invasive imaging with appropriate sedation or anaesthesia.*
Comment: The cumulative effect of repeated sedation or anaesthesia, and removal from the water, would have to be taken into account for studies involving serial imaging. These may enter the ‘moderate’ category depending upon the number and frequency of imaging sessions.
*Administration of substances by subcutaneous, intramuscular or intraperitoneal (intracoelomic) routes, gavage and intravenously via superficial blood vessels, where the substance has no more than mild impact on the animal, and the volumes are within appropriate limits for the size and species.*
Comment: The subcutaneous route is probably not feasible in fish; intravenous administration in fish is more difficult and invasive than in mammals. Few administration volumes have been defined for fish; see CCAC.^18^
*Superficial procedures, such as small superficial biopsies and non-surgical implantation of small transponders.*
*Application of external telemetry devices that cause only minor impairment to the animals or minor interference with normal activity and behaviour.*

The guidance in this document differs from the EC report in that it takes account of identification methods such as marking and tagging, because in fish these can be sufficiently invasive to contribute to the cumulative severity of the procedure, even if the tagging is conducted for husbandry purposes. In such cases, the impact on the animal should be taken into account, regardless of the different purposes of the procedures. For example, the introduction of a coded wire tag (CWT) into the nose, dorsal fin or other cartilagenous area has the potential to cause discomfort or pain. There are other issues associated with tagging aquatic species that can add to severity; for example, Carlin tags may be fouled by seaweed and shellfish, leading to increased mortality rates.^14^ In addition, the Working Group believes that there is a need for more research into the effects of commonly used marking methods such as removal of the adipose fin, which may have greater consequences than commonly believed.^15^

The EC report also contains many examples of procedures that are not commonly conducted on fish. There are also a number of procedures, such as electrofishing for capture^16^ that are not used in mammals and where the severity of the technique is likely to vary considerably between species and is also dependent upon the equipment used.

### Subthreshold

The examples in Table 1 are considered to fall below the lower threshold for regulation under the EC Directive. The EC Expert Group Report defines this as follows:

The lower threshold is exceeded if the animals may experience a level of pain, suffering or distress equivalent to, or higher than that caused by the introduction of a needle. Furthermore, the administration of anaesthesia for scientific purposes (excluding euthanasia) will bring a procedure above the lower threshold. Other types of lower thresholds are necessary for determining equivalence for specific research procedures … It is important to note that applying several such (‘below threshold’) techniques together in one animal may require the procedure to be classified as mild or higher.^3^

### Mild

The examples in Table 2 correspond to the ‘mild’ category in the EC Directive. The EC Expert Group Report defines this as follows:

Procedures on animals as a result of which the animals are likely to experience short-term mild pain, suffering or distress. Procedures with no significant impairment of the wellbeing or general condition of the animals.^3^

### Moderate

Table 3 lists examples within the ‘moderate’ category in the EC Directive, defined by the EC Expert Group Report as:

Procedures on animals as a result of which the animals are likely to experience short-term moderate pain, suffering or distress, or long-lasting mild pain, suffering or distress. Procedures that are likely to cause moderate impairment of the wellbeing or general condition of the animals.^3^

**Table 3:** Moderate procedures using fish

Prolonged removal of fish from water for the purpose of inducing stress. The impact on the fish is likely to vary between species, depending upon a number of factors such as their tolerance of handling and low oxygen levels and of course the level of stress required for the protocol. The Working Group suggests that this procedure would generally be moderate, although under different circumstances it could be classified as mild or severe.
‘Shaking’ of fish in a net out of water to cause a stress response.
Disease studies where the disease in question is known to cause death, but where the study can be controlled so that mortality does not occur – but where there is significant departure from normal health without it being prolonged or seriously compromising the fish.
Urine collection by insertion of a catheter into the bladder and attachment with appropriate suture material around the cloaca under anaesthesia.
Cannulation of blood vessels followed by successive blood sampling within acceptable limits for blood removal, that is where no significant physiological adaptation, or anaemia, will be caused.
Blood sampling where volumes are greater than those recommended by published guidelines and/or national legislation, or where sampling techniques may cause more than mild adverse effects.
Intraperitoneal injection of substances known to cause adhesions.
Fin clipping in conditions where infection is likely to occur, for example in warmer water (in cold water adapted species), or removal of substantial parts of a fin, or removal of part of a functionally important fin.
Removal of scales in order to promote fungal growth.
Intramuscular or intraperitoneal implantation of telemetry devices by surgical procedures (under general anaesthesia).
External attachment of telemetry devices where there is a risk of interference with normal activity and behaviour.
**Examples from the EC Working Group, with comments on their applicability to fish**
*Use of metabolic cages involving moderate restriction of movement over a prolonged period (up to 5 days)*
Comment: This translates as restriction of movement which interferes with normal activities over a significant but not prolonged period for fish.
*Evoking escape and avoidance reactions where the animal is unable to escape or avoid the stimulus, and which are expected to result in moderate stress.*
*Studies with modified diets that do not meet all of the animal's nutritional needs and are expected to cause moderate clinical abnormality within the time-scale of the study.*
*Breeding of genetically altered animals which are expected to result in a phenotype with moderate effects.*
*Frequent exposure to test substances which produce moderate clinical effects, and withdrawal of blood samples (>10% of blood volume) in a conscious animal within a few days without volume replacement.*
Comment: See comment in Table 2 with respect to total blood volumes.^18^
*Surgery under general anaesthesia and appropriate analgesia, associated with post-surgical pain, suffering or impaired general condition.*
*Acute dose-range finding studies, chronic toxicity/carcinogenetic tests, with non-lethal endpoints.*

### Severe

The examples in Table 4 would be ‘severe’ according to the EC Directive, defined by the EC Expert Group Report as:

Procedures on animals as a result of which the animals are likely to experience severe pain, suffering or distress, or long-lasting moderate pain, suffering or distress. Procedures that are likely to cause severe impairment of the wellbeing or general condition of the animals.^3^

**Table 4:** Severe procedures using fish

High stocking densities where significant physical harm occurs.
Severe restriction of movement which interferes with normal activities over a prolonged period.
Saltwater/freshwater challenge for scientific purposes (outside of normal species-appropriate husbandry procedures) where it cannot be predicted that the fish will adapt without severe effects or mortality.
Infections with a prolonged disease course, in which substantial loss of condition or other overt clinical signs, which cause a significant and prolonged departure from normal health, are required for the purposes of the study.
Disease studies where the disease in question is known to cause death and where the study cannot be controlled to avoid mortality.
Methods of marking fish that cause increased mortality or significant interference with normal behaviour, such as some jaw tags.
**Examples from the EC Working Group, with comments on their applicability to fish**
*Complete isolation for prolonged periods of social species.*
*Vaccine potency testing characterized by persistent impairment of the animal's condition, progressive disease leading to the animal's death, or associated with long-lasting moderate pain, distress or suffering.*
*Breeding animals with genetic disorders that are expected to experience severe or persistent impairment of general condition.*
*Surgical and other interventions in animals under general anaesthesia which are expected to result in severe or persistent moderate postoperative pain, suffering or distress, or severe and persistent impairment of the general condition of the animal.*
*Toxicity testing where death is the endpoint, or fatalities are to be expected and severe pathophysiological states are induced.*
*Forced swimming tests with exhaustion as the endpoint.*
Comment: The severity of forced exercise appears to be less in fish than mammals, because (i) an inescapable stimulus (electric shock) is generally used to make mammals run and (ii) fish appear to ‘opt out’ of the exercise at an earlier stage than mammals do. This procedure is therefore likely to be moderate in fish.

### Upper threshold

Table 5 lists examples of procedures that the Working Group believes would fall above the upper threshold. According to the EC Expert Group Report:

The upper threshold is exceeded if the animals may experience severe pain, suffering or distress which is likely to be long-lasting and cannot be ameliorated.^3^

Article 55 of Directive 2010/63/EU permits Member States that deem it necessary to allow the use of a procedure that exceeds the upper threshold to adopt a provisional measure to allow the procedure.^2^ The Member State must then inform the Commission, which will put the matter before a Committee which will decide whether to permit the provisional measure or require it to be revoked. The EC Working Group Report did not provide any examples of procedures above the upper threshold.

**Table 5:** Upper threshold procedures using fish

Pathophysiological studies of disease in which late characterization of the host–pathogen interaction is required, such that animals will experience substantial pain, suffering or distress which is long lasting.
Description of survival curves after infection with a pathogen. Note that some diseases can cause fish to rapidly deteriorate and die, in which cases the study would not fall above the upper threshold.
Description of survival curves or similar tests (for example after exposure to a chemical entity) where death is an endpoint and where death is preceded by prolonged and substantial pain, suffering or distress.

## Conclusion

Many procedures that are commonly performed on terrestrial laboratory animals have very different welfare implications when applied to fish, partly because of the inherent difficulties in the capture and handling of aquatic species. This, coupled with the biological variation exhibited in the large number of fish species involved, and our limited understanding of their welfare requirements, makes it difficult to offer detailed guidelines for classifying the severity of procedures on fish. Many common procedures are undoubtedly a greater challenge in fish than in terrestrial animals: these include (but are not limited to) marking, blood sampling, anaesthesia and analgesia.

The examples in this document are intended to facilitate severity classification, be an aid to discussion and to stimulate further research in this area. It will be up to the individual regulator, researcher and animal welfare officer to ensure that the 3Rs are fully implemented, including humane endpoints, regardless of the category in which a procedure has been placed.

## References

[1] Russell WMS, Burch RL The Principles of Humane Experimental Technique. London: Methuen & Co Ltd, 1959

[2] Council of the European Union *Proposal for a Directive of the European Parliament and of the Council on the Protection of Animals Used for Scientific Purposes*. Directive 8869/10 Brussels: Council of the European Union, 2010 See http://ec.europa.eu/environment/chemicals/lab_animals/proposal_en.htm (last checked 16 December 2010)

[3] Expert Working Group on Severity Classification Criteria *Expert Working Group on Severity Classification of Scientific Procedures Performed on Animals: Final Report*. Brussels: European Commission, 2009 See http://ec.europa.eu/environment/chemicals/lab_animals/pdf/report_ewg.pdf (last checked 16 December 2010)

[4] Participants at the International Consensus Meeting, Gardermoen, 22–24 September 2009 *Harmonisation of the Care and Use of Fish in Research* Oslo: Norecopa, 2009 See http://www.norecopa.no/norecopa/vedlegg/Consensus-sep09.pdf (last checked 16 December 2010)

[5] Norecopa *Tasks Needed to Increase Implementation of the 3Rs in Fish Research* Oslo: Norecopa, 2010 See http://www.norecopa.no/norecopa/vedlegg/Fish-tasks-prioritert.pdf (last checked 16 December 2010)

[6] Pickering AD Rainbow trout husbandry: management of the stress response. Aquaculture 1992;100:125–39

[7] Wendelaar Bonga SE The stress response in fish. Physiol Rev 1997;77:591–625 9234959 10.1152/physrev.1997.77.3.591

[8] Brydges NM, Boulcott P, Ellis T, Assessment of stress responses induced by different handling methods in three species of fish. Appl Anim Behav Sci 2009;118:137–43

[9] Young PS, Cech JJ Physiological stress responses to serial sampling and confinement in young-of-the-year striped bass, *Morone saxatilis*. Comp Biochem Physiol A, Comp Physiol 1993;105:239–44

[10] Pottinger TG, Calder GM Physiological stress in fish during toxicological procedures: a potentially confounding factor. Environ Toxicol Water Qual 1995;10:135–46

[11] Reed B, Jennings M *Guidance on the Housing and Care of Zebrafish (Danio rerio)* Southwater: RSPCA Research Animals Department, 2010 See http://rspca.org.uk/zebrafish (last checked 18 April 2011)

[12] Working Group appointed by Norecopa and The Research Council of Norway *Fish in Research – Environmental Requirements and Welfare Indicators for Fish. A Review of Research Needs* Oslo: The Research Council of Norway, 2009 See http://tinyurl.com/3dpvzox (last checked 19 April 2011)

[13] Lund V, Mejdell CM, Röcklinsberg H, Expanding the moral circle: farmed fish as objects of moral concern. Dis Aquat Org 2007;75: 109–18 17578250 10.3354/dao075109

[14] Strand R, Finstad B, Lamberg A, The effect of Carlin tags on survival and growth of anadromous Arctic charr, *Salvelinus alpinus*. *Environ Biol Fishes* 2002;64:275–80 See http://www.springerlink.com/content/n66j74t878536603 (last checked 17 December 2010)

[15] Reimchen TE, Tempel NF Hydrodynamic and phylogenetic aspects of the adipose fin in fishes. *Can J Zool* 2004;82:910–16 See http://web.uvic.ca/~reimlab/adipose.pdf (last checked 17 December 2010)

[16] Snyder DE *Electrofishing and its Harmful Effects on Fish: Information and Technology Report USGS/BRD/ITR-2003-0002* Denver, CO: US Government Printing Office, 2003 See http://electrofishing.net/wp-content/uploads/2009/01/snyder2003.pdf (last checked 17 December 2010)

[17] Home Office *Guidance Note: Water and Food Restriction for Scientific Purposes* London: Home Office, undated. See http://tinyurl.com/299zqjo (last checked 17 December 2010)

[18] Canadian Council on Animal Care (CCAC) *Guidelines on the Care and Use of Fish in Research, Teaching and Testing* Ottawa: CCAC, 2005 See http://www.ccac.ca/en/CCAC_Programs/Guidelines_Policies/GDLINES/Fish/Fish_Guidelines_English.pdf (last checked 17 December 2010)

